# Optimal Design of the Gating and Riser System for Complex Casting Using an Evolutionary Algorithm

**DOI:** 10.3390/ma15217490

**Published:** 2022-10-25

**Authors:** Bo He, Yiyu Lei, Mengqi Jiang, Fei Wang

**Affiliations:** Research Center of High-Temperature Alloy Precision Forming, School of Materials Science and Engineering, Shanghai University of Engineering Science, Shanghai 201620, China

**Keywords:** sand casting, multi-objective optimization, gating and riser system, evolutionary algorithm

## Abstract

The gating and riser system design is essential for both quality and cost in large-scale casting and is expected to reach several objectives simultaneously. However, even with the help of commercial simulation software, the design of gating and riser systems is still the result of a long-term trial-and-error optimal process owing to the conflict between the objectives. Several evolutionary algorithms (EAs) have been reported to be helpful in the selection of the geometrical dimensions of gating and riser systems. In this study, a route with sequential use of a multi-objective EA and single-objective optimization algorithm was developed to help design gating and riser systems, respectively. This route was applied in an actual case and verified using commercial simulation software. The results showed a dramatic decrease in the time cost in design and acceptable casting quality. Thus, the proposed design method is time-saving.

## 1. Introduction

The design of gating and riser systems directly affects the casting quality. Traditional design methods are based on long-term and expensive “trial-and-error” approaches. With the development of numerical simulation software, the design of gating and riser systems has shifted to a “proof-of-concept” approach [1]; however, it remains a long modification process that depends on the experience of the designer [2,3,4]. To balance quality and cost, an initial prototype of the gating and riser system that is close to its final optimal version should be set up to reduce the time spent on the design procedure.

The dimensions of the gating and riser systems are strongly related to both the quality and cost of casting. In terms of the riser, which is relatively simple and focuses mainly on material spending, single-objective optimization algorithms, such as the genetic algorithm (GA) and evolutionary topology algorithm, have been successfully applied to determine the geometric dimensions [5,6,7]. The design of a gating system is characterized by multiple conflicting objectives and involves many parameters, which is a typical multi-objective optimization problem [8]. However, multi-objective optimization faces objective conflict, in which no feasible solutions allow simultaneous optimal solutions for all objectives [9]. The classical approach to solve the multi-objective optimization problem is to simplify it to a single-objective optimization problem to use a simpler algorithm. Dučić et al. [10] used a GA to optimize the gating system design to obtain the minimum filling time. Tong et al. [11] employed a fruit fly optimization algorithm for gating system design based on simulation technology.

The multi-objective evolution algorithm (MOEA) is an ideal tool for solving multi-objective optimization problems by naturally producing multiple solutions called Pareto solutions [12]. The Pareto front obtained by the MOEA can provide a trade-off between objectives and support decision-makers in choosing the final optimal solution [13]. Kor [14] proposed an optimization strategy for the gating system using the elitist non-dominated sorting GA II (NSGA-II) [15], in which the objectives included no shrinkage porosity, minimized flow velocity, and maximized casting yields. However, expressing the shrinkage porosity mathematically is difficult; consequently, the individual fitness in NSGA II cannot be evaluated. The authors used simulation results instead of mathematical methods to solve the problem, and six optimized designs were obtained after 320 rounds of simulations. This strategy has a high requirement for computing power and incurs a large time cost when casting becomes complex.

In this study, an optimization route was developed in which the NSGA-II and GA were sequentially used on the gating and riser, respectively. A mathematical model was established for the gating design, consisting of three optimization objectives and three constraint functions. Because each optimization objective can be calculated in code, a set of Pareto optimal solutions can be automatically calculated by NSGA-II with the help of constraint functions, and the final design of the gating system is selected from the Pareto front based on the properties of the casting. The evolutionary algorithm (EA) toolbox Geatpy was used to run the optimization algorithms. The results show that the proposed method can achieve extremely high efficiency by obtaining a reasonable gating and riser system design.

## 2. Optimization Methods

**Figure 1:**
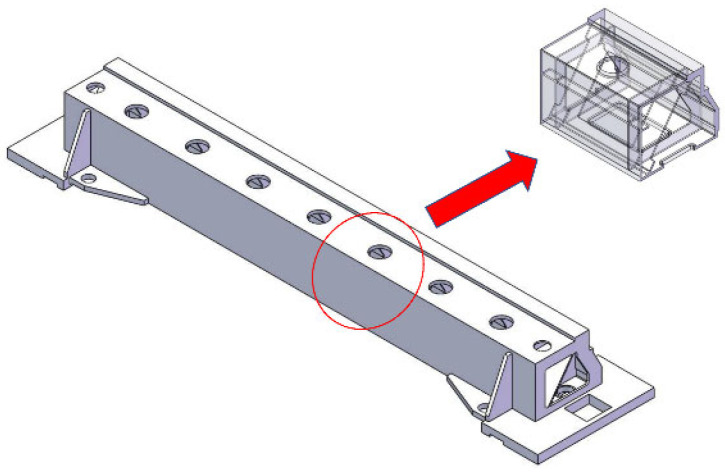
CAD model of the beam.

In this study, sand casting, which is the beam of the laser engraving machine, was made of ZL104 aluminum alloy (7% Si, 0.5% Mg, 0.15% Ti, 0.2% Fe), and the net mass was 91.6 Kg. The thermal properties of the ZL104 alloy are listed in Table 1. The computer-aided design (CAD) model in Figure 1 shows that the casting was characterized using a large-sized complex structure (545 mm × 2487 mm × 287 mm).

**Figure 2:**
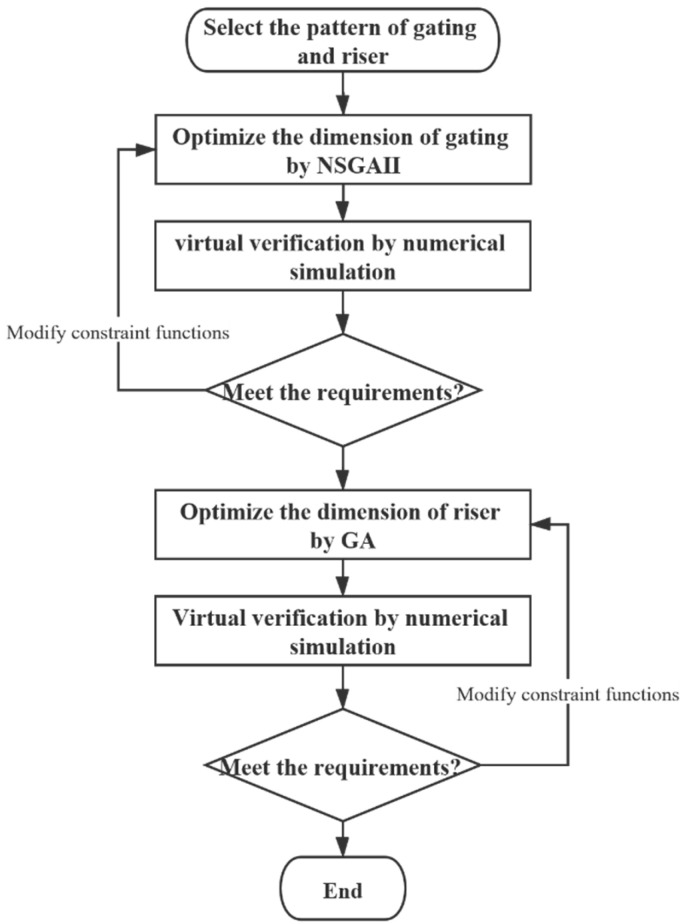
Optimization process flow.

The mold filling process of liquid metal has a great influence on the casting quality. A short filling time is a crucial design objective. However, a fast liquid metal front causes the surface of the liquid metal to be folded over by surface turbulence [16]. Therefore, a short filling time and stable liquid flow are two conflicting objectives that must be considered simultaneously. A reasonable design should be selected according to the actual situation. The casting yield is another optimization objective that cannot be neglected. Therefore, we attempted to design a gating to obtain a short filling time, high casting yield, and stable liquid metal flow, consisting of a three-objective optimization problem. The entire optimization process is illustrated in Figure 2.

In our optimization process, NSGA II was utilized to generate Pareto-optimal solutions for the gating system and the geometry of the gating system, such as the ratio of the choke section and the height of the sprue, were selected as decision variables in the mathematical models. The solution of this stage was determined based on the casting and Pareto front properties. The riser was relatively simple and only the casting yield required optimization; therefore, we considered it a single-objective problem. The single-objective evolution algorithm (GA) was employed in this stage. The numerical simulation software ProCAST was used to locate hotspots and evaluate the design quality. The SolidWorks CAD package provided the surface and volume data for the mathematical models.

### 2.1. Mathematical Models Set Up

#### 2.1.1. Gating System Setup

**Figure 3:**
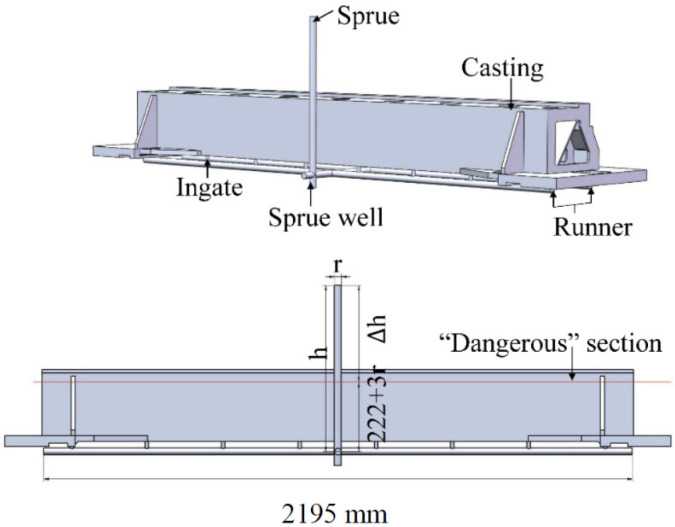
Gating system initialization along with decision variables.

An open bottom-filling gating pattern was selected for low turbulence. Figure 3 shows the initial design of the gating system, along with the decision variables of the radius of the choke section (r) and the distance from the choke section to the liquid top (h). According to the foundry manual, the cross-sectional area ratio of the gating system was set to A _sprue_/A _runner_/A _ingate_ = 1:2:4, and the dimensions of the remaining components of the gating system were defined based on the decision variables.

The “dangerous” section in Figure 3 is characterized by a minimum wall thickness section and a vertical plane that needs to limit the rising speed of liquid metal to meet the high filling performance requirements. Δh is the distance from the “dangerous” section to the top of the liquid. As the height of the in-gate was set to two times the diameter of the in-gate, the distance from the “dangerous” section to the bottom of the beam was 222 mm, and Δh was calculated as (h-222-3r).

#### 2.1.2. Mathematical Model of Gating System

Objective function. The design of the gating system is regarded as a minimum optimization problem with three minimum objectives by taking the reciprocal of the casting yield Y, as shown in Equation (1).

(1) Minimize f(x)=[T(x)Re(x)1Y(X)]

where ${x=[r,\;h]}^{T}$.

The filling time t can be simplified into the ratio of the volume of the casting V_CAST_ to the flow of liquid metal Q through the choke section without considering the energy and pressure loss during the filling process, as shown in Equation (2). The metal liquid flow state in the in-gate can be expressed by the Reynolds number [17], as shown in Equation (3).

(2) $$t=\frac{V_{\mathrm{CAST}}}{Q}=\frac{V_{\mathrm{CAST}}}{{\pi r}^{2}\sqrt{2\mathrm{gh}}}$$

(3) Re=vdv

where v is the velocity of the liquid metal flow (mm/ss), *v* is the dynamic viscosity of the liquid metal for aluminum alloy castings (*v* = 0.675 Pa.s), and d is the characteristic length (mm); for the internal pipe flow, d = 2r. The characteristic length for a non-internal pipe flow is given by Equation (4).

(4) $$d=\frac{4(\sec\mathrm{tion}\;\mathrm{area})}{\mathrm{wetted}\;\mathrm{perimeter}}=\frac{4A}{L}$$

The casting yield Y is defined as in Equation (5).

(5) $$Y=\frac{V_{\mathrm{cast}}}{V_{\mathrm{cast}}{+V}_{\mathrm{gating}}{+V}_{\mathrm{riser}}}$$

Constraint function. The Reynolds number corresponding to the rapid increase in inclusion was selected as the critical Reynolds number of the cavity, and the critical Reynolds number of the aluminum alloy casting was 2600 [18]. The maximum velocity of the liquid metal in the cavity can be calculated using Equation (3). According to Campbell’s rules [19], the maximum velocity v_Max_ is approximately 50 cm/s for most liquid metals and can be calculated using Equation (6).

(6) $$v_{\mathrm{Max}}=\frac{{4\mathrm{ARe}}_{\mathrm{Max}}v}{L}\;<\;50\;\mathrm{cm}/s$$

The design of the gating system should ensure that the cavity is tabled ultimately to prevent misruns, cold laps, and other defects. The filling performance can be guaranteed to avoid the above defects when the velocity of the liquid metal in the cavity is larger than the minimum velocity under certain conditions. The average minimum velocity of the liquid metal in the cavity can be calculated using Equation (7) [18].

(7) $$v_{\min}=0.22\times \frac{\sqrt{h_{a}}}{\delta_{a}\ln\frac{T}{380}}$$

where v_min_ is the average minimum velocity of the liquid metal along the cavity (cm/s); h_a_ and $\delta_{a}$ are the casting height and thickness (cm), respectively; and T is the pouring temperature (°C).

The actual liquid metal flow velocity of the “dangerous” section should be larger than v_min_ to avoid defects. The actual velocity v_a_ of the liquid metal front can be calculated using the following equation:

(8) $$v_{a}=\frac{Q\sqrt{\Delta h}}{A\sqrt{h}}$$

where Q is the liquid flow (mm^3^/s), A is the cross-sectional area (mm^2^), Δh is the distance from the section to the liquid top (mm), and h denotes the decision variable.

The constraint function shown in Equation (9) limits the search space for feasible solutions and ensures the quality of the casting.

(9) $$\mathrm{Subject}\;\mathrm{to}\;g\left(x\right)=\left[\begin{array}{c} v_{a}\left(x\right)\;<\;50\;\mathrm{cm}/s\\ v_{a}\left(x\right){\;>\;v}_{\min}\\ v_{a}\left(x\right){\;<\;v}_{\mathrm{Max}} \end{array}\right]$$

where ${x=[r,\;h]}^{T}$.

### 2.2. Mathematical Model of Riser

The design goal of the riser is to achieve a minimum volume while eliminating defects in the casting. The design of the riser is regarded as a single-objective minimum optimization problem and the objective function can be expressed as follows:

(10) $$\mathrm{Min}\;f\left(x\right){=V}_{r}\left(D,\;H,\;d,\;h\right)$$

where f(x) is the objective function and V_r_ is the minimum riser volume. To improve the feeding capacity of the riser, the design should obey the following rules:

(1) A feeding channel must exist between the feeding part in the casting and the riser until the casting is completely solidified;
(2) The riser should solidify at the same time or later than the casting;
(3) The riser should store sufficient liquid metal to compensate for the reduced volume of the casting due to solidification;
(4) The height of the riser neck should be greater than 15 mm and the riser should maintain its height.

Rule 1 requires the minimum diameter of the riser neck, which can be calculated using the cubic equation method [20] as follows:

(11) $$d_{r}=\sqrt[{3}]{A+B+\sqrt{{2\mathrm{AB}+B}^{2}}}+\sqrt[{3}]{A+B-\sqrt{{2\mathrm{AB}+B}^{2}}}+\sqrt[{3}]{A}$$

where $A={\left[\frac{k_{2}\left(1+\varepsilon \right)M_{c}}{{3k}_{1}}\right]}^{3}$, $B=\frac{{\varepsilon V}_{c}}{{2k}_{1}}$, K_1_ and K_2_ are the geometry parameters of the riser, Mc is the modulus of casting, V_c_ is the volume of the riser filled area in the casting, and ε is the volume shrinkage of metal solidification. For the cylinder riser, K_1_ = 0.25${\pi f}_{1},$ K_2_ = $\pi (0{.5+f}_{1})$, and f_1_ is a coefficient with a value of f_1_ = 1.5.

Rule 2 can be satisfied by Chvorinov’s heat-transfer criteria. Because there are nearly 20% potential errors when converting from modulus to freezing time, the modulus of the riser should be at least 1.2 times larger than the feeding part in the casting [21], as shown in Equation (12), where M_r_ and M_C_ are the moduli of the riser and feeding part in casting, respectively. For Rule 3, both the feeding efficiency of the riser and the solidification shrinkage rate of the liquid metal should be considered, which can be satisfied with the volume criterion, as shown in Equation (13).

(12) $$M_{r}\;>\;1{.2M}_{c}$$

(13) $$\left(\eta -\varepsilon \right)V_{r}{\;>\;\varepsilon V}_{c}$$

where η is the efficiency of the riser; for the cylinder riser, η = 0.14; ε is the liquid contraction during freezing; for the aluminum alloy, ε = 0.02; V_r_ is the volume of the riser; and V_c_ is the volume of the feeding part in the casting.

**Figure 4:**
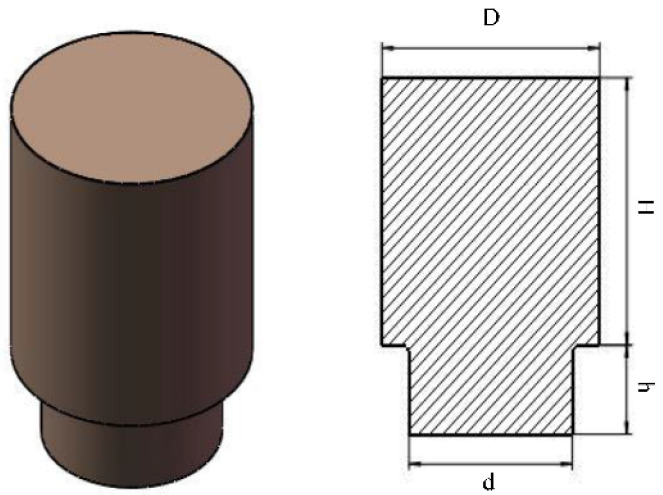
Riser CAD model with decision variables.

A cylinder riser is presented in this case and the CAD model and decision variables are shown in Figure 4. The objective function is expressed as follows:

(14) $${\mathrm{Minimize}\;V}_{r}=\frac{1}{4}\pi \left(D^{2}{H+d}^{2}h\right)$$

The constraint function is as follows:

(15) $$\mathrm{Subject}\;\mathrm{to}\;\left[\begin{array}{c} d\;\geq {\;d}_{r}\\ \left(\eta -\varepsilon \right)V_{r}{\;>\;\varepsilon V}_{c}\\ M_{r}\;\geq \;1{.2M}_{c}\\ H\in [D,1.5D]\\ h\in \left[15,d\right],\mathrm{if}\;d\;\geq \;30\;\mathrm{mm},h\in \left[15,30\right] \end{array}\right]$$

### 2.3. Initial and Boundary Conditions

The initial conditions in the numerical simulation include the material properties of each entity, pouring temperature, pouring time, and room temperature. The beam casting, gating system, and subsequent riser installation are made of ZL104 aluminum alloy. The resin sand in the material library is selected as the material for the sand box. The initial temperature of the liquid metal entering the pouring system is set at 710 °C and the pouring time is set by the concrete calculation results. The room temperature is set at 25 °C.

The boundary condition in numerical simulation is mainly the heat transfer coefficient of the contact surface between two different materials in the model. In this study, the heat transfer coefficient between the sand mold and casting, riser, and gating system was set at 500 W/(m^2^·K), the heat transfer coefficient between the sand mold and air at 20 °C was set at 10 W/(m^2^·K), and the heat transfer coefficient between the casting and cold iron was set at 2000 W/(m^2^·K). The heat transfer coefficient between the cold iron and the sand mold was taken as 1000 W/(m^2^·K). The preceding parameters are selected based on experience.

## 3. Results and Discussion

Table 2 lists the parameter values measured using SolidWorks software. The maximum velocity v_Max_ of the liquid metal in the cavity can be calculated as 93 mm/s using Equation (6) as Campbell’s rules. The minimum velocity v_min_ of the liquid metal was obtained as 24 mm/s using Equation (7). The actual velocity v_a_ of the liquid metal in the “dangerous” section can be determined by combining with Equation (8), as shown in Equation (16). The mathematical model is shown in Equation (17):

(16) $$v_{a}=\frac{Q\sqrt{\Delta h}}{A\sqrt{h}}=\frac{\pi r\sqrt[{2}]{2\mathrm{gh}}\sqrt{h-222-3r}}{57503\sqrt{h}}$$

(17) Minimize: f(x)=[T=(h+2×2195+9r)×πr2+34314252πr22gh1Y=(h+2×2195+9r)×πr234314252+1Re=2gh 2r0.675]Subject to: g(x)=[r2×h−222−3r>3117r2×h−222−3r<12077.]

where $\;x={\left[r,\;h\right]}^{T}$

The real number coding NSGA2 was selected, two-point crossover and roulette wheel selection were used as genetic operators, the crossover probability was set to 0.8, and the mutant probability was set to 0.3. The population range and generation number were set to 500 and the limits of the design variables were r = (1,50) and h = (300,900).

**Figure 5:**
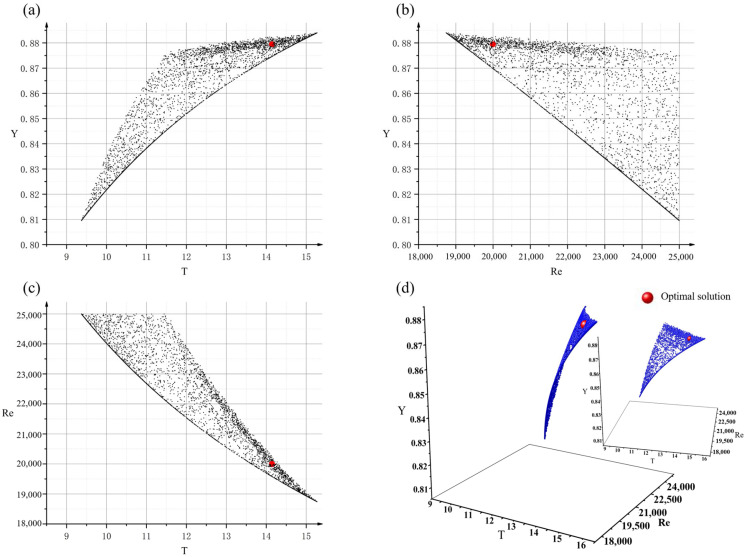
(**a**) Front view of the Pareto front, (**b**) left view of the Pareto front, (**c**) top view of the Pareto front, and (**d**) Pareto front obtained by NSGA II.

The Pareto front searched by the NSGA2 provided trade-off information between the three objectives, as shown in Figure 5. As observed from Figure 5a–c, the optimization objective T with the remaining two optimization objectives, Y and Re, were two sets of conflicting objectives. In other words, a higher yield tended to result in a more stable liquid metal flow and longer filling time. However, when the yield was unchanged, a shorter filling time was obtained by increasing the Reynolds number, as shown in Figure 5a,b.

In the Pareto front, the yield ranged between 80.1% and 88.4% without considering the riser system, the corresponding filling time ranged from 9.37 s to 15.27 s, and the corresponding Reynolds numbers ranged from 18,742 to 25,000. Because the constraint functions limit the search space of feasible solutions, all of the obtained nondominant solutions met the design requirements.

**Figure 6:**
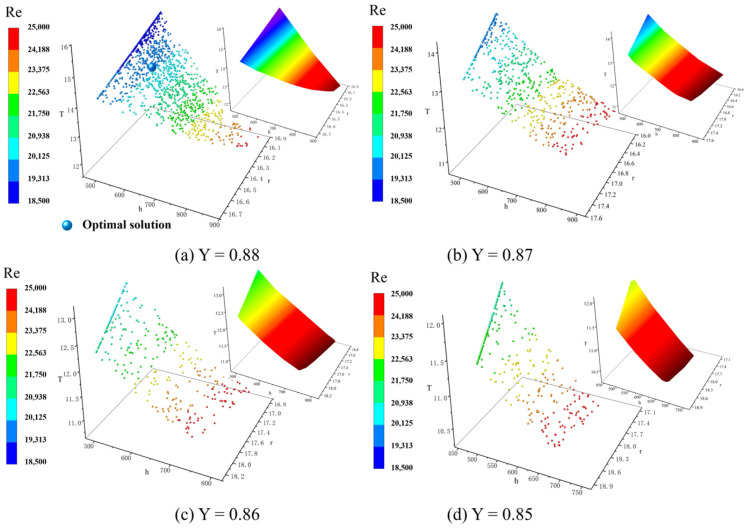
Trade-off between decision variables with filling time T and Reynolds number Re in the in-gate under specific yields Y: (**a**) Y = 0.88, (**b**) Y = 0.87, (**c**) Y = 0.86, (**d**) Y = 0.85.

Figure 6 shows the interaction effects of the gating system radios and gating system height on the liquid metal flow state and filling time in a specific casting yield (rounded down to the nearest integer). For higher yields, more optimal solutions were obtained, which means that the yield was a preferential objective in this mathematical model. Simultaneously, a higher yield results in higher economic benefits; therefore, in this case, the optimal solution was selected from Figure 6a with r = 16.28 mm and h = 550.10 mm, and the corresponding objective values were Y = 0.88, T = 14.14, and Re = 19,995.

**Figure 7:**
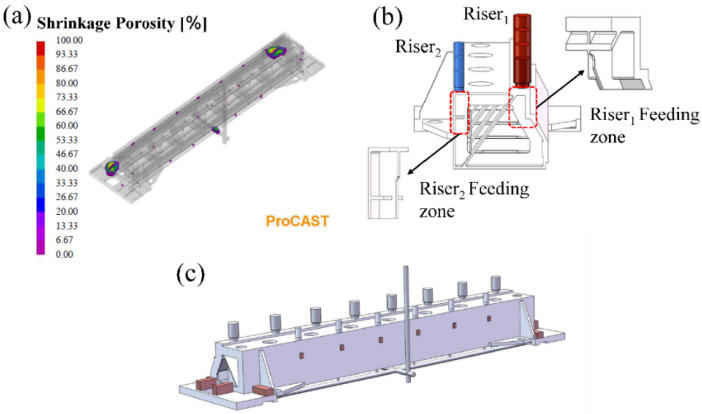
(**a**) Numerical simulation results of the beam with gating system, (**b**) location of the riser and feeding zone, and (**c**) optimal gating and riser system design with chill.

The distribution of the defects in the casting was determined using the numerical simulation software ProCAST, as shown in Figure 7a. As observed, the defects inside the casting were mainly distributed at the joints. Two sets of open cylinder risers of different sizes were placed on the casting top to eliminate top defects. The chills were placed on the lower part of the casting to construct a temperature field to form a bottom-to-top solidification, as shown in Figure 7c, and the thickness of the external chill was 1.2–1.5 times the thickness of the casting. The casting part fed by riser_2_ was the corner of two vertical planes with a thickness of 6 mm, as shown in Figure 7b, where the liquid metal in the casting and the riser solid quickly produced defects inside the casting. The safety factor α = 1.5 was set to riser_2_ to make the production more reliable.

The volume and area of the casting part fed by riser_1_ were V_1_ = 387,074 mm^3^ and A_1_ = 51,519 mm^2^, respectively, and those for riser_2_ were V_2_ = 77,025 mm^3^ and A_2_ = 25,671 mm^2^, respectively. Combined with Equation (15), the constraint function of riser_1_ and riser_2_ can be simplified to Equations (18) and (19), respectively.

(18) $${\mathrm{Riser}}_{1}:\;\left[\begin{array}{c} d\;\geq \;44\\ V_{r}\;>\;64,512\\ M_{r}\;\geq \;9\\ H\in \left[D,1.5D\right]\\ h\in \left[15,30\right] \end{array}\right]$$

(19) $${\mathrm{Riser}}_{2}:\;\left[\begin{array}{c} d\;\geq \;30\\ V_{r}\;>\;12,838\\ M_{r}\geq 5.4\\ H\in \left[D,1.5D\right]\\ h\in \left[15,30\right] \end{array}\right]$$

A real number coding GA was selected, two-point crossover and tournament selection was used as the genetic operator, and the population range was set to 300 with 500 generations. Table 3 lists the upper and lower limits of the design variables and optimization results of the riser design.

**Figure 8:**
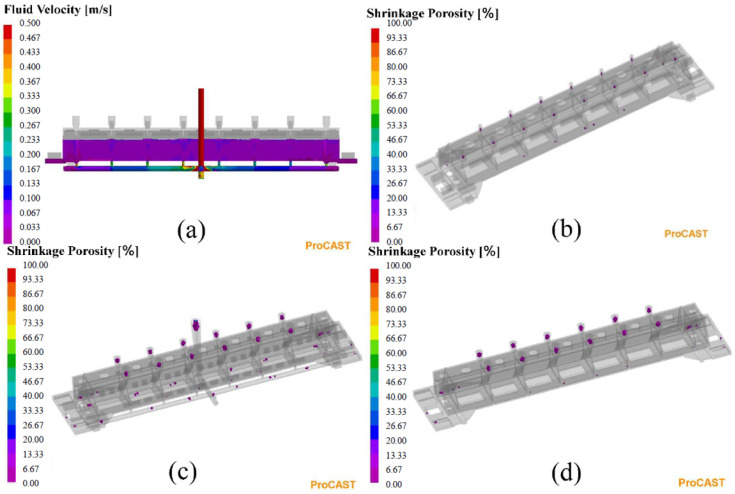
(**a**,**b**) Numerical simulation results of the optimal design case with the EA and (**c**,**d**) the traditional design case.

The simulation results in Figure 8a indicate that the velocity of liquid metal in the cavity was under 0.1 m/s, which was close to the maximum velocity of 93 mm/s in the constraint function. This indicates that the mathematical model established in this study is reasonable. From Figure 8b, the riser system eliminated the defects at the top of the casting and the casting quality was acceptable. As a comparison, Figure 8c,d show a result simulated based on a gating and riser system designed by the traditional method, setting the dimensions only by experience and manual. Table 4 shows the results of two different designs. Combining Figure 8 and Table 4, we find that the design with the EA can effectively improve the casting yield, reduce the time cost, and be closer to the optimal design than the traditional design method.

**Figure 9:**
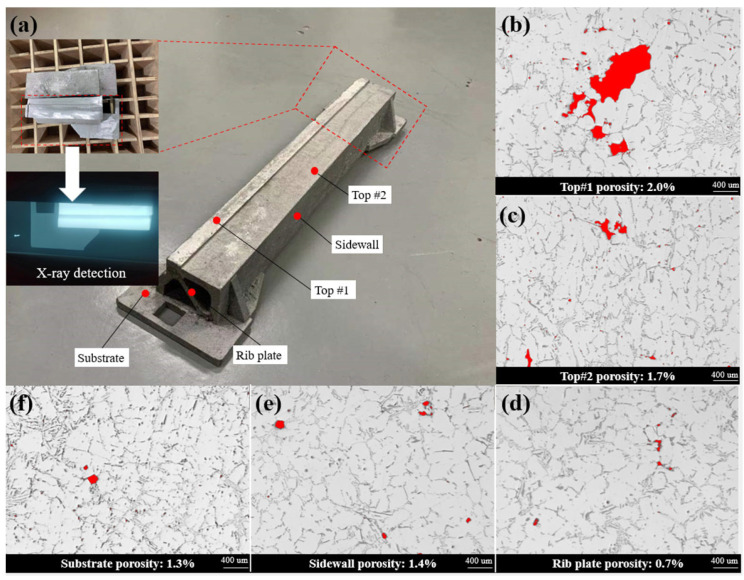
(**a**) Trial-produced beam casting and themicro-morphologies of different areas: (**b**) Top#1; (**c**) Top#2; (**d**) Rib plate; (**e**) Sidewall; (**f**) Substrate.

The proposed optimized solution of the feeder system was applied to the actual production, the pouring temperature was set to 710 °C, and the filling time was 4.5 s. The production results show that the optimized feeder system design can obtain an intact casting shape and a legible contour, as shown in Figure 9a. Samples were taken from the top region, sidewall, substrate, and rib plate of the casting, respectively; 20 images were taken for each sample using the optical microscope and the porosity of each sample was calculated with the image processing software ImageJ. As shown in Figure 9b,c, the porosity of the top region reached 2.0% and 1.7% and the pores were larger than the rest of the casting region. The main reasons for the relative high porosity in the upper region of the casting are the low static pressure and the floating of impurities in the casting process. In contrast, substrate and sidewall of the casting have higher quality. As shown in Figure 9e,f, the porosity of those region is 1.4% and 1.3%, respectively. The micro-topography of the rib plate is shown in Figure 9d; owing to the small thickness, the porosity of the rib plate is only 0.7%. An entity containing the above-mentioned features was cut out from the casting and X-ray detection was performed, as shown in Figure 9a, which indicated that the casting has a high quality with 98.6% relative density.

## 4. Conclusions

In this study, NSGA II and GA were used to design the gating and riser system, respectively. The mathematical model was proven to be effective through a study case. The conclusions of this study are as follows.

(1) Compared with the traditional design method, the use of an EA to design the gating and riser system can reduce the time cost and obtain a higher casting yield and acceptable casting quality design. With the increase in casting complexity and numerical simulation difficulty, the method proposed in this study can significantly reduce the time cost;
(2) The design of a gating system is a multi-objective optimization problem characterized by several conflicting objectives. The Pareto front obtained by NSGA II can provide complex trade-off information between objectives, providing decision-makers with a more flexible range of choices;
(3) The method proposed is particularly suitable for the gating and riser system design of complex thin-wall casings because of the dramatic decrease in time spent. It also makes further automatic design feasible.
(4) The trial casting has an intact shape and a legible contour, the casting relative density reaches 98.6%, and the X-ray detection shows the casting has a high quality, which verifies the effectiveness of the method proposed.

## Figures and Tables

**Table 1:** The thermal properties of the ZL104 alloy.

Density/ Kg/m^3^	Solidus Temperature/ °C	Liquidus Temperature/ °C	Thermal Conductivity/ W/(m^2^·K)	Critical Solidification Ratio	Solidification Shrinkage Volume Ratio/%
2630	555	595	137.53	0.7	7.14

**Table 2:** Model parameter values measured by SolidWorks.

Casting		Dangerous Section	
VCAST/mm^3^	34,314,252	Δh/mm	222+3r
T/°C	710	A/mm^2^	57,503
$\delta_{\alpha }$/cm	0.8	L/mm	12,202

**Table 3:** Limits of design variables and optimization results.

Limits of Design Variables				
	D/mm	d/mm	H/mm	h/mm
Riser_1_	[44, 100]	[44, 80]	[44, 150]	[15, 30]
Riser_2_	[30, 100]	[30, 60]	[44, 150]	[15, 30]
Optimization results				
Riser_1_	57.89	45	81	15
Riser_2_	33	31.5	50.25	15

**Table 4:** Results of the traditional design method and design with the EA.

	Design with EA				Traditional Design Method			
	r/mm		h/mm		r/mm		h/mm	
Sprue	16.28		550		20		651	
Runner	16.28		L = 2195		20		L = 2195	
Ingate	8.14		32.56		10		40	
Riser_1_	D/mm	d/mm	H/mm	h/mm	D/mm	d/mm	H/mm	h/mm
	57.89	45	81	15	60	45	80	19
Riser_2_	33	31.5	50.25	15	40	55	70	16
Material spending amount/kg	108.5				122.3			
Casting yield/%	84.4				74.9			
Time cost/h	27				60			

## Data Availability

The CAD model used in this study was provide by a third party. Direct request for the material may be made to the provider as indicated in the Acknowledgements. All data and codes generated or used during the study are available from the corresponding author by request. NSGA Ⅱ python code with a mathematical model. GA python code with a mathematical model.
